# Supplementary material for: Evolutionary and functional insights into the mechanism underlying body-size-related adaptation of mammalian hemoglobin
Source: eLife. 2019 Oct 24;8:e47640. doi: 10.7554/eLife.47640 (PMC6812962; doi:10.7554/eLife.47640)
Supplement: Supplementary file 1. — aThe MWC allosteric parameters for Hb evolutionary datasets reported in this table were obtained as described in Rapp and Yifrach (2017). The KT and KR parameters correspond to the affinity of oxygen to the T and R Hb MWC quaternary states, respectively, whereas c and L correspond to the relative affinity (c = KR/KT) and conformational ratio (L = [T]/[R]) of the T and R states, respectively. bThe mammalian Hb species analyzed in the current analysis, as reported in Milo et al. (2007). cHill coefficient at half-saturation calculated based on Equation 5 and using the KR, KT, and L MWC parameters of the different mammalian Hb binding curves (see Rapp and Yifrach, 2017). dHill coefficients at half-saturation obtained upon fitting Hb oxygen saturation data to the Hill equation (Equation 1), as reported in Milo et al. (2007). [file elife-47640-supp1.docx]

**Supplementary File 1**

| **Supplementary File 1. MWC allosteric parameters of the hemoglobin evolutionary dataset oxygen saturation curves^a^** | | | | | | | |
| --- | --- | --- | --- | --- | --- | --- | --- |
| **#** | *^b^***Mammalian species** | ***K*_R_**  **(mmHg)** | ***K*_T_**  **(mmHg)** | *^a^****c* x(10^-3^)** | *^a^****L* x(10^5^)** | *^c^***Calculated**  ***n*_H_^MWC^** | **Observed**  *^d^****n*_H_^Hill^** |
| 1 | *African elephant* | 1.0±0.2 | 120.2±2.5 | 8.1±1.3 | 1530.5±166.3 | **2.9±0.2** | **2.88±0.12** |
| 2 | *Horse* | 1.4±0.5 | 112.2±26.2 | 12.3±5.6 | 0.7±0.6 | **2.8±0** | **2.76±0.02** |
| 3 | *Camel* | 2.6±0 | 72.4±0.2 | 36.3±0.5 | 0.1±0 | **2.3±0.1** | **2.26±0.05** |
| 4 | *Cow* | 0.2±0 | 89.1±0.1 | 2.2±0.3 | 1565.4±9.7 | **2.9±0.1** | **2.87±0.05** |
| 5 | *Asian elephant* | 0.3±0.2 | 114.8±11.1 | 2.8±1.4 | 240.3±92.5 | **3±0.2** | **3.01±0.16** |
| 6 | *Mole* | 0.7±0 | 91.2±0.6 | 8±0.3 | 9.5±0.3 | **2.7±0.1** | **2.68±0.1** |
| 7 | *Orangutan* | 0.7±0.2 | 120.2±11.3 | 5.6±1.8 | 16.6±6.2 | **2.8±0.1** | **2.76±0.09** |
| 8 | *Gorilla-F* | 0.1±0.1 | 93.3±0.3 | 1.5±0.6 | 7457.4±86.7 | **2.7±0.2** | **2.72±0.16** |
| 9 | *Chimpanzee* | 0.7±0.1 | 95.5±2.1 | 7.6±0.9 | 12±1.1 | **2.9±0.1** | **2.87±0.08** |
| 10 | *Platypus* | 1.3±0.3 | 158.5±27.8 | 8.3±2.5 | 1.3±0.9 | **3.2±0** | **3.18±0.04** |
| 11 | *Human* | 1.1±0.1 | 125.9±4.6 | 8.5±0.9 | 2173.4±480.4 | **2.8±0.2** | **2.87±0.04** |
| 12 | *Antelope* | 0.5±0.5 | 338.8±145 | 1.5±1.6 | 83.4±142.8 | **3.6±0.2** | **3.59±0.15** |
| 13 | *Goat* | 0.4±0.5 | 436.5±196.4 | 0.9±1.1 | 274.5±494.2 | **3.5±0.1** | **3.54±0.11** |
| 14 | *Dog* | 0.8±0 | 104.7±0.3 | 8.1±0.4 | 9.2±0.1 | **2.6±0.1** | **2.62±0.06** |

*^a^*The MWC allosteric parameters for Hb evolutionary datasets reported in this table were obtained as described in Rapp and Yifrach (2017). The *K*_T_ and *K*_R_ parameters correspond to the affinity of oxygen to the **T** and **R** Hb MWC quaternary states, respectively, whereas *c* and *L* correspond to the relative affinity (c= *K*_R_/ *K*_T_) and conformational ratio (*L*= [**T**]/[**R**]) of the **T** and **R** states, respectively.

*^b^*The mammalian Hb species analyzed in the current meta-analysis, as reported in Milo et al (2007).

*^c^*Hill coefficient at half-saturation calculated based on **Equation 5** and using the *K*_R_, *K*_T_, and *L* MWC parameters of the different mammalian Hb binding curves (see Rapp and Yifrach (2017)).

*^d^*Hill coefficients at half-saturation obtained upon fitting Hb oxygen saturation data to the Hill equation (**Equation 1**), as reported in Milo et al (2007).
